# TIGIT, a novel immune checkpoint therapy for melanoma

**DOI:** 10.1038/s41419-023-05961-3

**Published:** 2023-07-26

**Authors:** Wei Tang, Jun Chen, Tianlong Ji, Xiufeng Cong

**Affiliations:** 1 Department of Neurosurgery, Shengjing Hospital of China Medical University, Shenyang, 110000 Liaoning Province China; 2 Department of Oncology, Shengjing Hospital of China Medical University, Shenyang, 110000 Liaoning Province China; 3 Department of Radiation Oncology, The First Hospital of China Medical University, Shenyang, 110000 China

**Keywords:** Tumour immunology, Skin cancer

## Abstract

Melanoma is the most aggressive and deadliest type of skin cancer. In the last 10 years, immune checkpoint blockades (ICBs) including PD-1/PD-L1 and CTLA-4 inhibitor has been shown to be effective against melanoma. PD-1/PD-L1 and CTLA-4 inhibitors have shown varying degrees of drug resistance in the treatment of melanoma patients. Furthermore, the clinical benefits of ICBs are also accompanied by severe immune toxicity. Therefore, there is an urgent need to develop new immune checkpoint inhibitors to optimize melanoma therapy and reduce cytotoxicity. T-cell immunoreceptor with immunoglobulin and immunoreceptor tyrosine-based inhibition motif domain (TIGIT) is thought to activate inhibitory receptors in T cells, natural killer (NK) cells, and regulatory T cells (Tregs), and has become a promising target for immunotherapy. Studies have found that TIGIT can be detected in different stages of melanoma, which is closely related to the occurrence, development, and prognosis of melanoma. This review mainly describes the immunosuppressive mechanism of TIGIT and its role in antitumor immunity of melanoma, so as to provide new ideas and schemes for the clinical treatment of melanoma with targeted TIGIT.

## Facts

1. TIGIT binds CD155 and CD112 to create immune suppression; CD226 binds CD155 to deliver a positive signal; CD96 binds CD155 to create immune suppression.
2. TIGIT exerts inhibitory effects on innate and adaptive immunity through multiple mechanisms, including triggering T/NK cell-intrinsic inhibition, inducing immunosuppressive DCs, inhibiting CD226 signaling, enhancing immunosuppression of Tregs and promoting Fap2-induced T/NK cells inhibition.
3. Blockade of TIGIT on CD8^+^ T cells, Tregs, and NK cells augments antitumor immunity.

## Open question

1. How to find and intervene in targets involved in the immunosuppressive effect of TIGIT?
2. How to develop TIGIT blockers to restore antitumor immunity in melanoma?
3. What drugs can improve the efficacy and safety of TIGIT blockers in the treatment of melanoma?

## Introduction

Melanoma is the most aggressive and deadliest type of skin cancer. Worldwide, melanoma represents 1.7% of all newly diagnosed cancers, and 0.6% of cancer-related deaths [1]. The incidence of melanoma has doubled in the last 30 years [2]. The main cause of death in melanoma patients is the extensive spread of tumors to the liver, lung, brain, bone, lymphatic system, and other organs [3]. The most effective treatment for melanoma is surgery resection, and for unresectable metastatic melanoma, radiotherapy, and chemotherapy have traditionally been used. These last two therapies, however, have shown many inconveniences like resistance, secondary cancers, or toxicity to healthy tissues [4].

In the last ten years, immune checkpoint blockades (ICBs) including PD-1/PD-L1 and CTLA-4 inhibitor, designed to restore immune cell activity against pathogens and cancer cells, has been shown to be effective against many types of cancer [5]. Currently, PD-1/PD-L1 inhibitors are approved by the FDA for the treatment of more than a dozen tumors, including melanoma [6]. However, ~40%–65% of melanoma patients have intrinsic resistance to PD-1-based therapy [7, 8], and 43% of responders develop secondary resistance within 3 years [9]. In addition, anti-CTLA-4 antibody has also been used in the clinical treatment of melanoma [10]. Unfortunately, anti-CTLA-4 has a low clinical response rate for melanoma [11]. Furthermore, the clinical benefits of ICBs are also accompanied by severe immune toxicity, including cardiotoxicity [12], pneumonia [13], hepatitis, colitis [14], pancreatitis [15], and endocrine dysfunction [16]. Additionally, other new ICBs have also been approved, such as anti-LAG3 in melanoma [17].

As T cells and natural killer (NK) cells are central parts of the immune system [18, 19], an increasing number of studies have focused on the inhibitory immune checkpoints they express on their surfaces. T-cell immunoreceptor with immunoglobulin and immunoreceptor tyrosine-based inhibition motif domain (TIGIT) is thought to activate inhibitory receptors in T cells, NK cells, and regulatory T cells (Tregs), and has become a promising target for immunotherapy [18, 20, 21]. Studies have found that TIGIT can be detected in different stages of melanoma [22], which is closely related to the occurrence, development, and prognosis of melanoma [23, 24]. However, it is not known whether TIGIT-based immunotherapy could induce better treatment results and less toxicity compared to anti-PD-1/PD-L1 or anti-CTLA-4 immunotherapy in melanoma, which needs to be further explored. This review mainly describes the immunosuppressive mechanism of TIGIT and its role in antitumor immunity of melanoma, so as to provide new ideas and schemes for the clinical treatment of melanoma with targeted TIGIT, and discusses whether anti-TIGIT may be an alternative to anti-PD-1/PD-L1 or anti-CTLA-4.

## TIGIT structure and ligands

TIGIT, also known as WUCAM [20], Vstm3 [25], VSIG9 [26], is a co-inhibitory receptor belonging to the immunoglobulin superfamily [27] that was first identified in 2009 [21]. TIGIT is composed of an extracellular immunoglobulin variable region (IgV) domain, a type 1 transmembrane domain, and an intracellular domain containing an immunoreceptor tyrosine-based inhibitory motif (ITIM) and an Ig tail-tyrosine (ITT)-like motif constitute [21, 27–29]. The TIGIT molecule is relatively conservative, and the amino acid sequence of human TIGIT shares 88%, 67%, and 58% homology with those of rhesus monkeys, dogs, and mice, respectively [21].

TIGIT is expressed on activated traditional αβ T cells, but also on memory T cells, Tregs, follicular helper cells, and NKT cells [30, 31]. In addition to T cells, TIGIT is also expressed in NK cells [27], which is induced in mouse NK cells and constitutively expressed in human NK cells. For cancers, compared to CD226, TIGIT is weakly expressed by naive T cells and co-expressed with PD-1 on mouse and human tumor-infiltrating CD8^+^ T cells [32, 33]. Additionally, TIGIT is highly expressed in peripheral blood Tregs of healthy donors and cancer patients and is further upregulated in the tumor microenvironment [34]. Studies have shown that the hypomethylation of TIGIT loci is a characteristic of human Treg, and the expression of TIGIT makes the activated Treg better different from the activated effector T cells in vitro or in vivo [35]. The ligands of TIGIT include CD112, and poliovirus receptor (PVR), of which PVR is the high-affinity cognate receptor of TIGIT, and PVR is also known as CD155, Necl-5, and Tage4 [36]. Furthermore, TIGIT competes for ligands with CD226 (DNAM-1) and CD96 (TACTILE) [37]. Studies have shown that TIGIT can effectively block the binding of CD155 and CD96 [38] or CD226 [39], which further proves that TIGIT has the highest affinity for CD155 (Table 1). Notably, TIGIT not only competes with CD226 for ligands, but also can directly cis-bind to CD226 to prevent its homo-dimerization, so that CD226 cannot bind to CD155 to play a co-stimulatory role. However, the degree of co-expression of TIGIT and CD226 on T cells in the inflamed tissue is still unclear.

**Table 1:** Ligand-binding affinities for TIGIT, CD226, and CD112R.

Ligand/receptor affinity	TIGIT	CD226	CD112R	CD96
CD155	1–3 nM	114-199 nM	/	37.6 nM
CD112	Not measurable	0.31–8.97 µM	88 nM	/

CD155 is mainly expressed on the surface of dendritic cells (DCs), T cells, B cells, and macrophages, and also in nonhematopoietic tissues such as the kidney, nervous system, and intestine to varying degrees [31]. In addition, CD155 has been reported to be highly expressed in a variety of human malignancies, including melanoma [40], pancreatic cancer [41], colon cancer [42], lung adenocarcinoma [43], and glioblastoma [44]. CD155 is a cell adhesion molecule that affects cell proliferation, migration, invasion, and adhesion through tumor-associated signaling pathways. It also interacts with CD226, TIGIT, and CD96 on immune cells, affecting the function of tumor-infiltrating T cells and NK cells [45]. In melanoma patient, high CD155 expression in tumors is also associated with resistance to anti-PD-1 therapy [46]. Braun et al. also revealed that CD155 on melanoma cells drives resistance to immunotherapy by inducing degradation of the activating receptor CD226 in CD8^+^ T cells [47]. These data suggest that CD155 expression in tumors has a dual pro-tumor effect, both tumor-intrinsic and through inhibition of antitumor immunity.

**Fig. 1:**
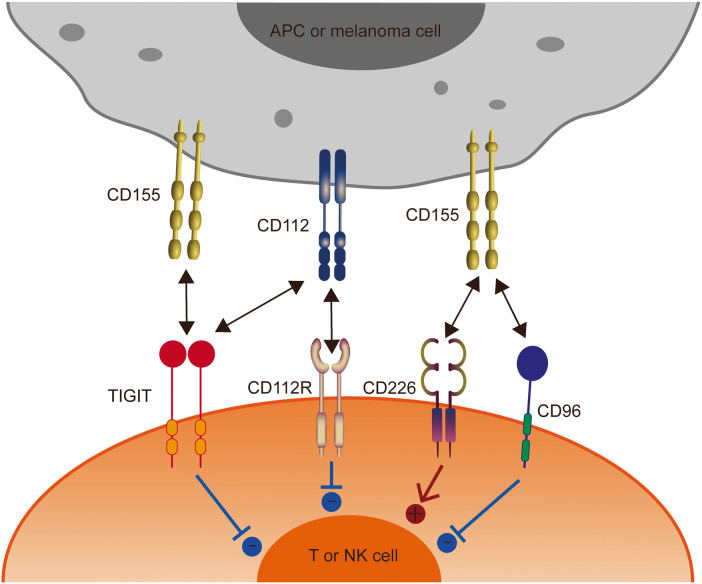
The interaction of TIGIT family receptors and ligands. TIGIT, CD226, CD96, and CD112R are expressed in T cells and NK cells. The ligands CD155 and CD112 are expressed on tumor cells or APCs. TIGIT delivers inhibitory signals by binding to CD155 and CD112, with the highest affinity for CD155. CD226 and CD96 compete with TIGIT for binding to CD155, but with lower affinity than TIGIT. CD226 delivers activating signals. However, whether CD96 triggers inhibitory or activating signals remains to be determined. CD112R and CD226 also competitively bind to CD112, with higher affinity with CD112R. APCs, antigen-presenting cells.

CD112 is expressed on DCs and monocytes [48] and is highly expressed in various cancers [49–51], but is rare in melanoma cell lines [51]. Furthermore, CD112 has a higher affinity for CD112R (PVRIG) than for TIGIT, and suppresses T cells mainly via binding to CD112R [52, 53] and not via TIGIT [53]. However, in melanoma, whether TIGIT works primarily by binding to CD155 rather than CD112 requires more direct evidence, which needs to be further explored in the future. Details of TIGIT structure and ligands are shown in Fig. 1.

## TIGIT in cancer progression

TIGIT overexpression has been found in the cellular microenvironment of several cancers and is associated with a poor prognosis for cancer, including melanoma [54, 55].

In aggressive breast cancer, a large-scale transcriptome data analysis found that TIGIT was highly specifically expressed in aggressive breast cancer, and its pro-tumor activity was associated with immune-related genes. TIGIT expression was positively correlated with gene expression related to inflammation and immune response 33721026. Therefore, TIGIT expression appears to be strongly associated with advanced malignant pathological types of breast cancer and may be a potential biomarker of breast cancer progression. In renal cell carcinoma, immunohistochemical and flow cytometry results showed that TIGIT expression in cancer tissues was increased compared with adjacent cancer, but the number of TIGIT^+^T cells and TIGIT^+^NK cells was not related to clinicopathological features. In addition, high TIGIT expression was associated with the clinicopathological characteristics of lung adenocarcinoma, which was associated with advanced TNM staging, lymphoid metastasis, distant metastasis, and low expression of antitumor immunity-related genes [56]. Similarly, CD8^+^T-cell populations with high TIGIT expression in peripheral blood in patients with hepatocellular carcinoma were inversely correlated with overall survival (OS) and progression-free survival (PFS) [57]. Interestingly, increased TIGIT expression in gastric cancer appears to be a favorable event. TIGIT expression correlates with an active immune landscape, survival and immunotherapeutic sensitivity, and favorable prognosis. Patients with high TIGIT expression respond better to immunotherapy than those with low TIGIT expression [58].

In addition, in addition to the above-mentioned solid tumors, the high expression of TIGIT on immune cells also plays an important role in the progression of hematological tumors. An increase in the number of TIGIT-expressing CD4^+^ T cells and CD8^+^ T cells in tumors in patients with follicular lymphoma is associated with poor prognosis and survival [59]. In addition, high expression of TIGIT in peripheral blood CD8^+^ T cells in patients with acute myeloid leukemia is associated with the development of primary refractory disease [59].

These data suggest that TIGIT has an inhibitory effect in antitumor immunity in cancer patients, but whether TIGIT’s role in melanoma is different from other cancers is not clear, and it is worth further exploration in the future.

## The mechanisms of TIGIT co-inhibition

**Fig. 2:**
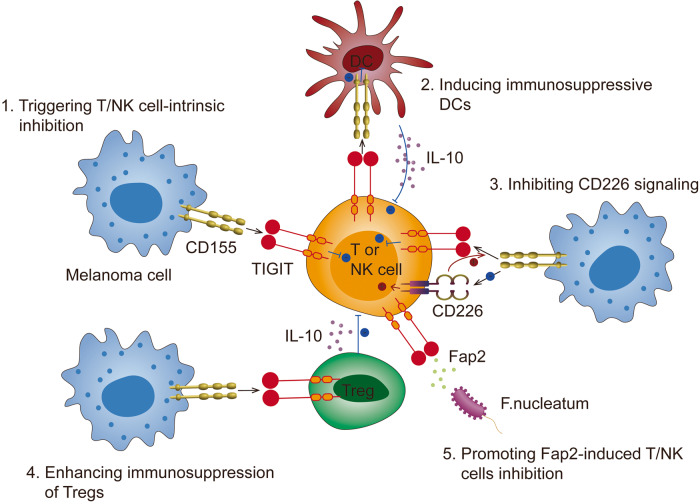
The mechanisms of TIGIT co-inhibition. TIGIT exerts inhibitory effects on innate and adaptive immunity through multiple mechanisms. 1. TIGIT binds CD155 to trigger T/NK cell-intrinsic inhibition; 2. TIGIT binds CD155 to induce immunosuppressive DCs; 3. TIGIT binds CD155 to inhibit CD226 signaling; 4. TIGIT binds CD155 to enhance immunosuppression of Tregs; 5. Fap2 protein from Fusobacterium nucleatum binds TIGIT to induce T/NK cell inhibition.

TIGIT exerts inhibitory effects on innate and adaptive immunity through multiple mechanisms, including triggering T/NK cell-intrinsic inhibition, inducing immunosuppressive DCs, inhibiting CD226 signaling, enhancing immunosuppression of Tregs and promoting Fap2-induced T/NK cells inhibition (Fig. 2).

### Triggering T/NK cell-intrinsic inhibition

At first, TIGIT on T cells can act directly on T cells by attenuating T-cell receptor (TCR) -driven activation signals [60]. Specifically, TIGIT can inhibit the expression of TCR itself by inducing downregulation of the TCR-α chain and the molecules that make up the TCR complex, thereby inhibiting the proliferation and activation of CD8^+^ T cells [60]. In addition, TIGIT can reduce TCR-induced p-ERK signaling in CD8^+^ T cells [61]. However, no studies have confirmed that TIGIT plays a role in melanoma immune evasion by directly downregulating TCR signaling.

In NK cells, upon binding of TIGIT to CD155, the ITT-like motif in the tail is phosphorylated at Tyr225 and binds to the cytoplasmic adaptor Grb2, which recruits SH domain-containing inositol-5-phosphatase (SHIP1) to inhibit PI3K and MAPK signaling cascades, thereby downregulating NK cell activity [28]. Phosphorylated ITT-like motifs also bind to β-arrestin2, follow by recruiting SHIP1 to disrupt TRAF6 autoubiquitination, thereby inhibiting NF-κB activity and IFN-γ production in NK cells [29].

### Inducing immunosuppressive DCs

In addition to directly inhibiting T cells, Tigit on T cells can also bind to CD155 on DCs to indirectly inhibit the activation of T cells. TIGIT induces phosphorylation of CD155 on DCs, thereby enhancing interleukin (IL)-10 production and diminishing IL-12 production by dendritic cells [21]. However, whether this DC-dependent indirect regulation exists in the immune response of melanoma remains to be explored in the future.

### Inhibiting CD226 signaling

As TIGIT competes with CD226 for CD155 ligands, blocking CD226-mediated T cells and NK cells co-stimulation helps promote immunosuppression by TIGIT [62]. Inozume et al. reported that TIGIT upregulation and CD226 downregulation of melanoma-specific cytotoxic T lymphocytes (CTLs) were induced by tumor stimulation. These findings suggested that an imbalance in CD226 and TIGIT expression is a novel mechanism of T-cell suppression in the effector phase of the antitumor CTL response [63]. Further, CD226 blockade abrogates the effects of dual PD-1 and TIGIT blockade on the proliferation and cytokine production of tumor antigen-specific CD8^+^ T cells in melanoma [32]. In addition, TIGIT can prevent co-stimulatory signaling via CD226 by blocking CD226 homo-dimerization [33].

### Enhancing immunosuppression of Tregs

Tregs express a variety of inhibitory receptors that support their inhibitory function, including TIGIT. TIGIT is highly expressed by a subset of natural Tregs in mice [34] and the majority of Tregs in humans [34, 64, 65]. Notably, studies have found that TIGIT^+^ Tregs were more inhibitory than TIGIT^-^ Tregs in melanoma patients [64, 65]. Interestingly, Fourcade et al. [64] showed that in melanoma patients, Tregs showed increased expression of TIGIT and decreased expression of the competitive co-stimulatory receptor CD226 compared with CD4^+^ effector T cells, resulting in an increased TIGIT/CD226 ratio. A high TIGIT/CD226 ratio in Tregs correlates with increased Treg frequencies in tumors and poor clinical outcome upon ICBs. The future challenge lies in determining whether the TIGIT/CD226 ratio in Tregs can be used as a biomarker of clinical response to ICB in melanoma patients.

### Promoting Fap2-induced T/NK cells inhibition

Bacteria, such as Fusobacterium nucleatum, are present in the tumor microenvironment of various cancers, including melanoma [66]. *F. nucleatum* has been shown to directly interact with TIGIT in NK and T-cells through its Fap2 protein to inhibit NK cell cytotoxicity and suppress T-cell activity [67]. Although it has not been demonstrated whether this mechanism is involved in immune escape in melanoma, the possibility of such a mechanism is not excluded due to the presence of *F. nucleatum* in melanoma.

## TIGIT in melanoma immunotherapy

TIGIT is expressed on human tumor-infiltrating CD8^+^ T cells, NK cells, Th, and Treg cells in melanoma [32, 63]. Decreased TIGIT expression in CD8^+^ T cells was associated with inhibition of tumor growth in melanoma cells [68]. Lee et al. evaluated the expression of TIGIT in 124 melanoma patients by immunohistochemistry and analyzed their clinicopathological features and survival. The results showed that high expression of TIGIT was associated with worse survival. These results suggest that TIGIT has an inhibitory effect on antitumor immunity in melanoma patients [69]. In the following sections, we will discuss which tumor-infiltrating immune cell populations are inhibited by TIGIT to cause immune escape from melanoma and possible therapeutic strategies (Table 2).

**Table 2:** TIGIT in melanoma immunotherapy.

TIGIT-expressing immune cells	Treatment strategies	Mechanism	Ref.
CD8^+^T cells	Elraglusib (9-ING-41)	Reducing TIGIT expression on CD8^+^ T cells	[68]
CD8^+^T cells	Dual PD-1/TIGIT blockade	Enhancing the proliferation and function of tumor antigen-specific CD8^+^ T cells	[32, 63]
CD8^+^T cells	Dual PD-1/TIGIT blockade	Promoting the activation of CD226 signaling pathway	[80]
CD8^+^T cells	Dual anti-CD96/TIGIT	Restoring melanoma-infiltrating CD8^+^ T-cell antitumor immunity	[83]
Tregs	Activating CD226 in Tregs together with TIGIT blockade	Counteracting Tregs suppression	[64]
Tregs	Dual anti-TIM-3/TIGIT	Reducing immunosuppression of Tregs	[86]
NK cells	IL-15 stimulation together with TIGIT blockade	Augmenting antitumor immunity of NK cells	[95]
NK cells	Dual anti-CTLA-4/TIGIT	Improving the immunosuppression of NK cells against melanoma	[96]
NK cells	Deletion of CISH	Optimizing NK cell killing properties and decreasing TIGIT immune checkpoint receptor expression	[98]

### Blockade of TIGIT on CD8^+^ T cells augments antitumor immunity

CD8^+^ T cells can not only kill tumor cells immediately by secreting factors such as granzyme B, perforin, and INF-γ, but also generate immune memory and reside in peripheral tissues to maintain antitumor immune response and inhibit tumor growth [70]. Thus, augmenting the CD8^+^ T-cell antitumor response is a major strategy in most cancer immunotherapies [71]. Blocking TIGIT in the co-culture system of melanoma cells and CD8^+^ T cells in vitro restored the production of IFN-γ in CD8^+^ T cells [72]. In addition to anti-TIGIT mAb, a recent clinical trial found that Elraglusib (9-ING-41) also reduced TIGIT expression on CD8^+^ T cells, thus exerting an inhibitory effect on melanoma [68]. Elraglusib is a reversible ATP-competitive small-molecule inhibitor of glycogen synthase kinase-3β, a serine/threonine kinase with multiple roles in tumor growth, cell invasion, and metastasis [73–75]. Nevertheless, single TIGIT blockade achieved no or moderate antitumor efficacy in experimental tumor models [33, 76–78] and in enhancing in vitro functionality of human tumor-infiltrating CD8^+^ T cells [79]. Similarly, dual PD-1/TIGIT blockade also enhanced the proliferation and function of tumor antigen-specific CD8^+^ T cells and CD8^+^ tumor-infiltrating lymphocytes (TILs) in melanoma patients compared with single TIGIT blockade [32, 63]. However, dual blockade of TIGIT and PD-1 should be further explored to induce potent antitumor CD8^+^ T cells responses in patients with advanced melanoma.

Notably, blocking CD226 in vitro and in a murine melanoma model nullifies the dual blocking effect of PD-1/TIGIT, suggesting that TIGIT blockade promotes CD155 binding to CD226 to activate CD8^+^ T-cell immune activity [32]. In addition, PD-1 inhibition rescued CD226 activity by preventing PD-1-SHP2 dephosphophorylation of the CD226 intracellular domain [80]. This indicates that dual PD-1/TIGIT blockade may enhance antitumor immunity by promoting the activation of CD226 signaling pathway. However, Chauvin et al. found that CD8^+^ TILs downregulated CD226 expression in melanoma, which may be an important barrier to limit the dual blocking effect of PD-1/TIGIT in melanoma patients [32]. Since CD155 plays a critical role in mediating the downregulation of CD226 expression on melanoma-infiltrating immune cells [81], reducing CD155 expression in melanoma may be a potential strategy to enhance the dual blocking effect of PD-1/TIGIT on melanoma [82].

Besides PD-1 blockade, other ICBs combined with TIGIT blockade also enhance antitumor immune responses in melanoma. For example, Mittal et al. [83] observed that CD96 co-expressed with TIGIT in CD8^+^ melanoma TILs, and dual anti-CD96/TIGIT combination therapy was superior to anti-TIGIT monotherapy in suppressing tumor growth and improving mouse survival in B16F10 melanoma. Further study also found that anti-PD-1 combined with CD96/TIGIT dual blockade on melanoma growth inhibition effect is significantly better than the dual anti-CD96/TIGIT combined treatment. This provides a new strategy for restoring melanoma-infiltrating CD8^+^ T-cell antitumor immunity by blocking TIGIT.

### Blockade of TIGIT on Tregs augments antitumor immunity

Tregs, as an important mechanism for regulating homeostasis of the immune system and the immune tolerance of the body, play an important role in tumor immune escape [84]. In contrast to the effects on CD8^+^ T cells, TIGIT expression on Tregs enhanced the suppressor function of Tregs [85]. In melanoma, activation of CD226 opposes TIGIT to disrupt the suppression and stability of Tregs [64], which provide the rationale for novel immunotherapies to activate CD226 in Tregs together with TIGIT blockade to counteract Treg suppression in melanoma patients. Additionally, Kurtulus et al. [86] revealed that TIGIT^+^ Tregs upregulated the expression of the co-inhibitory receptor TIM-3 in tumor tissues. Then, a TIGIT-null melanoma mouse model was constructed and anti-TIM-3 was found to have a higher survival rate than observed with TIGIT deficiency alone, suggesting that TIM-3 and TIGIT synergized to suppress antitumor immune responses in melanoma. These results support the combined use of ICBs targeting Tregs in melanoma immunotherapy.

### Blockade of TIGIT on NK cells augments antitumor immunity

NK cells are derived from bone marrow lymphoid stem cells, their differentiation and development depend on the bone marrow and thymus microenvironment, and are mainly distributed in bone marrow, peripheral blood, liver, spleen, lung, and lymph nodes [87]. Different from T and B cells, NK cells are a type of lymphocyte that can non-specifically kill tumor cells and virus-infected cells without prior sensitization [88]. NK cell-based cancer immunotherapy, which refers to the activation of NK function and showing substantial therapeutic effects on tumors [89], is increasingly used in melanoma [90].

Single-cell characteristics of the melanoma cell landscape identified the high expression of TIGIT on tumor-infiltrating NK cells [91], offering new options for clinical translation. The signal balance between co-stimulatory and co-inhibitory signal molecules expressed in NK cells regulates the immune activity of NK cells [92]. Consistent with CD8^+^ T cells, CD226 is an activating receptor, while TIGIT and CD96 are inhibitory receptors that bind to tumor-derived CD155 to regulate NK cell-mediated tumor immunotherapy [93]. Notably, NK cell-based therapies represent a powerful approach to kill MHC class I-deficient tumors that may arise upon CD8^+^ T-cell-mediated immune destruction of MHC class I-presenting tumor cells [94]. Chauvin et al. [81] found that membrane-bound CD155 triggers CD226 internalization and degradation in NK cells, while IL-15 promoted increased expression of TIGIT and CD226 on tumor-infiltrating NK cells in melanoma. The study further revealed that IL-15 stimulation together with TIGIT blockade promotes NK cell-mediated destruction of MHC class I-deficient melanoma, while CD226 blockade decreases the effects of IL-15 and TIGIT blockade. In addition, another study also showed that CD155 inhibits the CD226-mediated cytotoxic activity of NK cells, thus promoting the lung colonization of B16/BL6 melanoma [95].

Interestingly, other ICBs combined with TIGIT blockade also enhanced antitumor immune responses of NK cells in melanoma. For example, Rethacker et al. [96] found decreased CTLA-4 and TIGIT expression in blood NK cells from 16 patients who received ipilimumab, which is a fully humanized anti-CTLA-4 monoclonal antibody approved by FDA for late-stage melanoma [97], suggesting that the combination of CTLA-4 and TIGIT blockade may improve the immunosuppression of NK cells against melanoma. A recent study also found that the deletion of cytokine-inducible SH2-containing protein (CISH), a critical immune checkpoint, favors NK cell accumulation to the primary tumor, optimizes NK cell killing properties, and decreases TIGIT immune checkpoint receptor expression, limiting NK cell exhaustion [98]. This makes dual targeting of CISH and TIGIT a potential strategy to activate NK cell-dependent melanoma immunotherapy.

## Clinical application of anti-TIGIT monoclonal antibodies in melanoma

At present, anti-TIGIT monoclonal antibodies have been conducted in multiple clinical trials in melanoma, however, these clinical trials are still in the stage of recruiting patients, and the results of the study have not been reported. Details of the clinical trials that have been conducted are shown in Table 3.

**Table 3:** Clinical trials of anti-TIGIT monoclonal antibodies in melanoma.

Registration number	Study phase	Primary outcome measures	Arms and interventions	Research status
NCT05130177	II	ORR	Zimberelimab (anti-PD-1) plus Domvanalimab (anti-TIGIT)	Recruiting
NCT05665595	III	RFS	Pembrolizumab (anti-PD-1) plus Vibostolimab (anti-TIGIT)/Pembrolizumab	Recruiting
NCT04305054	II	ORR, AE	Pembrolizumab plus Vibostolimab/Pembrolizumab plus Quavonlimab (anti-CTLA-4)/Pembrolizumab+Favezelimab (anti-LAG3)/Pembrolizumab	Recruiting
NCT04305041	II	ORR, AE	Pembrolizumab plus Quavonlimab plus Vibostolimab	Recruiting
NCT05483400	II	pCR, ORR	Tiragolumab (anti-TIGIT) plus Atezolizumab (anti-PD-1)	Not yet recruiting
NCT04303169	II	pCR, AE	Pembrolizumab/Pembrolizumab plus Vibostolimab/Favezelimab plus Pembrolizumab	Recruiting
NCT05060432	II	ORR, AE	EOS-448 (anti-TIGIT) plus Pembrolizumab/Dostarlimab (anti-PD-1)	Recruiting

*RFS* recurrence-free survival, *ORR* objective response rate, *AE* adverse event; *pCR* pathological complete response.

## Conclusion and prospects

Prior to the introduction of immunotherapy for the treatment of advanced melanoma, outcomes were generally poor despite the application of many cytotoxic agents and combinations [99]. Melanoma is a highly malignant tumor, most of which are found at an advanced stage due to its rapid development and evolution, which makes the operation impossible [100, 101]. Therefore, it is necessary to further explore the application of immunotherapy in melanoma and explore the relevant mechanisms. TIGIT exerts inhibitory effects on innate and adaptive immunity through multiple mechanisms, including triggering T/NK cell-intrinsic inhibition, inducing immunosuppressive DCs, inhibiting CD226 signaling, enhancing immunosuppression of Tregs and promoting Fap2-induced T/NK cells inhibition. However, these mechanisms have not all been confirmed in melanoma immune response and need to be further explored in the future.

Decreased TIGIT expression in immune cells was associated with the inhibition of tumor growth in melanoma patients, making TIGIT a promising target in melanoma immunotherapy. Blockade of TIGIT on CD8^+^ T cells, Tregs, and NK cells augment antitumor immunity. However, single TIGIT blockade has minimal effects on melanoma growth in most experimental tumor models and is also insufficient to reinvigorate functions of human tumor-infiltrating CD8^+^ T cells. TIGIT blockade synergizes with PD-1/PDL-1 blockade or CD96 blockade to enhance antitumor CD8^+^ T-cell immunity in preclinical models. Additionally, dual targeting of CTLA-4 or CISH and TIGIT may be a potential strategy to activate NK cell-dependent melanoma immunotherapy. However, direct clinical and preclinical evidence is lacking. In addition, future studies need to carry out relevant clinical trials to compare the efficacy and toxicity of anti-TIGIT with anti-PD-1/PD-L1 or anti-CTLA-4 in melanoma. Furthermore, CD226 plays a critical role as a master regulator of dual PD-1/TIGIT blockade. Its downregulation by CD8^+^ T cells and NK cells in melanoma may represent a major obstacle to the success of dual PD-1/TIGIT blockade in the clinic. Therefore, it appears essential to design novel strategies to augment CD226 expression and signaling or prevent its downregulation in melanoma immunotherapy.

## Supplementary information

aj-checklist

## Data Availability

All data that support the findings of this study are available from the corresponding author upon reasonable request.
